# Hydrogels-Assisted Cell Engraftment for Repairing the Stroke-Damaged Brain: Chimera or Reality

**DOI:** 10.3390/polym10020184

**Published:** 2018-02-13

**Authors:** Daniel González-Nieto, Laura Fernández-García, José Pérez-Rigueiro, Gustavo V. Guinea, Fivos Panetsos

**Affiliations:** 1 Center for Biomedical Technology, Universidad Politécnica de Madrid, 28040 Madrid, Spain; laura.fernandezg@ctb.upm.es (L.F.-G.); jose.perez@ctb.upm.es (J.P.-R.); gustavovictor.guinea@ctb.upm.es (G.V.G.); 2 Departamento de Tecnología Fotónica y Bioingeniería, ETSI Telecomunicaciones, Universidad Politécnica de Madrid, 28040 Madrid, Spain; 3 Biomedical Research Networking Center in Bioengineering Biomaterials and Nanomedicine (CIBER-BBN), 28040 Madrid, Spain; 4 Departamento de Ciencia de Materiales, ETSI Caminos, Canales y Puertos, Universidad Politécnica de Madrid 28040 Madrid, Spain; 5 Neurocomputing and Neurorobotics Research Group: Faculty of Biology and Faculty of Optics, Universidad Complutense de Madrid, 28040 Madrid, Spain; fivos@ucm.es; 6 Instituto de Investigación Sanitaria, Hospital Clínico San Carlos Madrid, IdISSC, 28040 Madrid, Spain

**Keywords:** biomaterials, hydrogels, stem cells, stroke, brain repair

## Abstract

The use of advanced biomaterials as a structural and functional support for stem cells-based therapeutic implants has boosted the development of tissue engineering applications in multiple clinical fields. In relation to neurological disorders, we are still far from the clinical reality of restoring normal brain function in neurodegenerative diseases and cerebrovascular disorders. Hydrogel polymers show unique mechanical stiffness properties in the range of living soft tissues such as nervous tissue. Furthermore, the use of these polymers drastically enhances the engraftment of stem cells as well as their capacity to produce and deliver neuroprotective and neuroregenerative factors in the host tissue. Along this article, we review past and current trends in experimental and translational research to understand the opportunities, benefits, and types of tentative hydrogel-based applications for the treatment of cerebral disorders. Although the use of hydrogels for brain disorders has been restricted to the experimental area, the current level of knowledge anticipates an intense development of this field to reach clinics in forthcoming years.

## 1. Introduction 

In recent decades, considerable progress has been made in the development of experimental therapies to treat different brain disorders. Among the different approaches examined, cell therapy has been configured as a viable option for restoring the damaged brain. Tissue recovery after damage has been associated with the mobilization and integration of new cerebral cells derived from exogenous sources. Alternatively, endogenous brain repair mechanisms may also be stimulated by the transplantation of different cell populations with the ability to secrete factors that mobilize endogenous neurogenesis. Cell transplantation through a systemic intravascular route has been regularly tested in animal models and brain injury patients. Intra-venous and intra-arterial administrations are considered relatively feasible to perform and are less invasive than cerebral implantation. However, the systemic route is handicapped by the inefficient mobilization of therapeutic cells from the blood towards the brain tissue given that most cells are retained in the pulmonary capillaries, spleen, liver, and kidneys [1,2], therefore limiting the number of cells able to reach the brain by crossing the blood-brain barrier which separates the circulatory system from the brain tissue. Despite its higher invasiveness, cerebral implantation is the most promising strategy because of the possibility of grafting a greater number of therapeutic cells in the area of interest, which might be restricted to or near the area of the lesion [3,4,5,6,7,8]. The relevance of this approach has been supported in later years by the progressive expansion of the number of clinical trials performing intracerebral cell implants in patients with cerebral damage using different populations of stem cells and progenitors of embryonic, hematopoietic, and mesenchymal sources.

However, even the cerebral route is not free of difficulties. Among them, we should mention the reduced temporality of the graft due to the poor survival ratio of the cells as well as their dispersion towards regions far away from the area of interest, thus diluting any therapeutic effect. It is assumed that in response to injury the brain tissue is converted into a hostile microenvironment not only for the brain itself, but also for the grafting of different cell populations that generally do not survive beyond a few weeks after implantation [5,6,8]. The lack of survival factors combined with cell death signals from reactive astrocytes, microglia and peripheral leukocytes not only contribute to damage the still alive brain tissue in perilesional areas but most likely also constitute the main causes for poor cell engraftment. 

In regenerative neuroscience, the combined implantation of cells and different biomaterials to increase the viability of cellular grafts constitutes a very promising approach in full expansion [9]. In this context hydrogels rise as a powerful and versatile group of architectonic elements for cell encapsulation and brain reconstruction due to their special chemical and physical structures with stiffness modules in the range of the soft tissues such as the brain. Given the increasing variety of applications and experimental models for the development of biomaterials-based stem cell therapies for neurological disorders, this review focuses on current knowledge on the use of hydrogels-based cell therapy for ischemic brain injury (ischemic stroke), which represents the most frequent type of disabling neurological pathology [10]. An additional objective is to provide a global view of the biomedical problems as well as of the past and current trends in the related experimental and translational research. Finally, we aim at highlighting the opportunities, type of applications and possible benefits of hydrogels-based cellular therapies for ischemic brain injuries. For a deeper understanding of the application of hydrogels and therapeutic cells in the treatment of other less frequent but not less important neurological disorders, several comprehensive reviews have been included in the Reference Section [11,12,13,14].

## 2. Clinical Scenario and Challenges to Solve

Cerebrovascular diseases encompass a set of syndromes of different etiology and severity that lead to transient or permanent disorders of brain function. Pathologically, cerebrovascular diseases may be a consequence of: (i) alterations in cerebral blood flow; (ii) disturbances in blood circulation that modify blood flow and pressure; and (iii) disorders of cardiac function. Stroke represents a sudden onset of cerebrovascular disease and occurs in most cases through a vascular obstructive process (ischemic; ~85% of stroke patients) or by the rupture of one or more brain blood vessels and extravasation of blood (effusion) into the extravascular space (hemorrhagic; ~15% of stroke patients), both leading to a reduction or total abolition of blood supply to the brain. Epidemiologically, stroke is a leading cause of adult disability and cognitive impairment and is the second-leading global cause of death behind ischemic heart disease [10,15]. It is estimated that after stroke a substantial proportion of patients (25–50%) remain dependent for at least one daily task up to six months after injury [16] producing incalculable personal, family, and social costs worldwide. The main risk factors for stroke include high blood pressure, ischemic heart disease, diabetes mellitus, disorders of heart rhythm, high blood cholesterol and lipids, smoking, physical inactivity, diet and weight, family history, and genetic background. Due to the progressive increase in life expectancy, it is assumed that in the next few decades there will be a parallel increase in the number of stroke cases (confirmed by the fact that stroke incidence has increased by up to 20% in the last few years [15,17]) increasing the susceptibility of suffering this vascular affectation at an even earlier age [18].

Mitigation of stroke-originated brain damages strongly depends on the recognition of signs and symptoms in the hospital emergency area and the consequent rapidity of medical intervention. Hemiparesis, loss of sensation, impaired speech, vertigo, and gait disturbances represent the initial clinical signs [19,20]. In ischemic stroke, the early reestablishment of blood flow in the obstructed vessel is a strong determinant for the clinical evolution of the patient. The intravenous injection of the tissue plasminogen activator (tPA) alone or in combination with surgical procedures such as endovascular thrombectomy for the recanalization of the occluded vessel are currently the main therapeutic approaches for eliminating the obstructive clot [19,21,22,23]. However, the percentage of stroke patients that can get benefit from these two treatments is extremely low. Due to the short therapeutic window (3.0–4.5 h after the onset of the attack) and the risk of complications, only 6% of the affected people are eligible for these treatments. In addition, tPA is effective in less than 10% of the treated cases, reducing the possible beneficiaries to less than 0.6% of the affected population. Endovascular thrombectomy is even less effective, being also very limited the number of teams specialized in this type of intervention.

Patients who survive a stroke and do not receive adequate treatment within this narrow therapeutic window or when its reception is ineffective often show disabilities with different degrees of affectation [24]. For these patients, no therapies are currently available for the repair of the damaged brain or for the promotion of a satisfactory degree of functional recovery [25]. Physical and/or cognitive rehabilitation, transcranial magnetic (TMS) or direct-current (tDCS) stimulation may lead to a functional improvement in a reduced number of patients during the chronic phase of disease. There are contradictory results among different groups on the efficacy of these types of approaches most likely related to the heterogeneity and low number of patients participating in the clinical trials and the lack of consensus in the methodologies used [26,27,28,29]. Instead of reconstructing the neural tissue, neuro-rehabilitation and training strategies promote the emergence of new circuitry in non-injured areas surrounding (perilesional) the damaged tissue. This perilesional tissue may take control of specific functions that were lost and initially dependent of the injured areas (“vicarious” function), as has been demonstrated previously in pioneering studies with monkeys and humans [30,31]. However, and despite contradictions [32], neuro-rehabilitation and training strategies may result very useful for the functional improvement of stroke patients particularly when applied in synergy with other promising therapeutic alternatives, as for example biomaterials-based therapy.

Cell therapy has been proposed as a strong and convenient strategy for the reconstruction of the damaged brain tissue or guide cerebral plasticity in perilesional areas enhancing functional recovery. Transplanted cells should modulate recovery mechanisms similar to those acting in spontaneously healing or rehabilitation improvements. The first objective of cell therapy is to provide a source of transplanted exogenous cells that can differentiate into the main components of nervous tissue (neurons, astrocytes, oligodendrocytes, and vascular endothelial cells). Additionally, transplanted cells should stimulate endogenous self-repairing mechanisms and produce biomolecules to promote neurogenesis and angiogenesis. A variety of stem cells, progenitors and differentiated cells have been engrafted in different models of brain damage. Since the first studies using cells from neural or hematopoietic origin to promote recovery in ischemic animals [33,34,35,36,37], in the last two decades, we have witnessed a growing number of clinical trials with different types of cell populations transplanted either systemically or intracerebrally [38,39,40,41,42,43,44,45,46,47]. In the next section, we discuss the timeline for stroke treatment after two decades of experimental and clinical research in cell-based therapies and we introduce elements for reasoning about the use of hydrogels based on different materials as a support for cell engraftment and function. 

## 3. Therapeutic Intervention after Stroke: What Have Two Decades of Cell Therapy Research Taught Us?

The use of cell therapies for the treatment of human diseases dates back to the 1950s, specifically in the area of hemopathies. In those years, two independent groups reported how the intravenous administration of hematopoietic stem cells and progenitors from spleen or bone marrow favored the hematopoietic regeneration of mice previously subjected to myeloablation by lethal irradiation [48,49]. Thomas et al. were pioneers in performing the first cell-based therapy by transplanting bone marrow in six human patients previously mieloablated with irradiation or chemotherapy to treat leukemia and multiple myeloma [50]. Since then, cell therapy has been configured as a plausible strategy for tissue and organ regeneration in other human disorders, mostly by using undifferentiated cells with characteristic self-renewal and potency properties, the so-called stem cells (SCs). In addition to bone marrow transplantation, which probably constitutes one of the most relevant cell-based therapies with proven efficacy in the treatment of malignant disorders related to hematopoietic dysfunctions, cell transplantation has been approved for other diseases such as the use of limbic stem cells (lSCs) to regenerate the cornea after ocular burns [51] or the use of mesenchymal stem cells (mSCs) to treat perianal fistulas in Crohn’s Disease [52]. Very recently transgenic SCs have been used clinically to produce skin implants for junctional epidermolysis bullosa disease [53]. The number of clinical studies has grown exponentially. For example in the case of mSCs more than 300 clinical trials have been carried out in the last years to evaluate the repair potential of this cell population in bone, cartilage, heart, lung, liver, kidney, autoimmune, gastrointestinal and neurological disorders [54]. However, despite great advances in preclinical and clinical environments, the market for cell-regenerative medicine is still in its infancy and no more than 10 marketing licenses have been granted in the European Union to treat diseases not related with cerebral disorders [55]. Another example of the slow transition to the regular use of SCs in patients is illustrated by the first approval by the US Food and Drug Administration (FDA) of a cell-based gene therapy to treat lymphoblastic leukemia on August 2017 [56].

In the area of cerebral disorders and specifically in stroke, during the last two decades, numerous studies with animals have validated the efficiency of different cell populations (mainly stem cells and progenitors of diverse tissue sources) in promoting functional recovery after brain injury. In addition, considerable progress has been made in the study of the molecular and cellular substrates responsible for this recovery. Knowledge obtained in last few years about the action mechanisms of the different SCs as well as about their therapeutic limitations moved this field into a new scenario, the combined implantation of SCs with different biomaterials of natural or artificial origin. Generally, different stem cells and progenitors (SCs/P) are capable to differentiate into progressively more mature cell phenotypes (neurons, astrocytes, and oligodendrocytes) as well as to secrete different trophic factors [57,58]. Both abilities can be exploited therapeutically: (i) to protect neurons from a hypoxic hostile environment and decrease the post-ischemia inflammatory response [59,60]; (ii) to favor the replacement (restitution) of damaged neurons with new neural cells creating new circuitry in the injured areas [8,61,62]; and (iii) to enhance tissue remodeling mechanisms in perilesional regions of the damaged brain. This last one is especially relevant for functional recovery after cerebral ischemia [3].

When a cerebral infarction occurs the injured area is anatomically and functionally categorized in a central nucleus where blood flow has been totally interrupted and brain tissue evolves towards infarcted condition (infarct core) and a perilesional area where the blood flow has be reduced but not abolished containing structurally intact but functionally inactive neural networks (ischemic penumbra) [63]. If the reduced blood flow persists, penumbra is irreversibly damaged while reperfusion with an adequate blood flow speeds down the ischemic process. After the damaged area has been established the perilesional tissue might undergo functional modifications (plasticity) to sustain post-stroke functional recovery as has been reported in humans subjects and animal models [64]. 

### 3.1. Stem Cells for Neuroprotection and Repair of the Injured Brain

**Figure 1:**
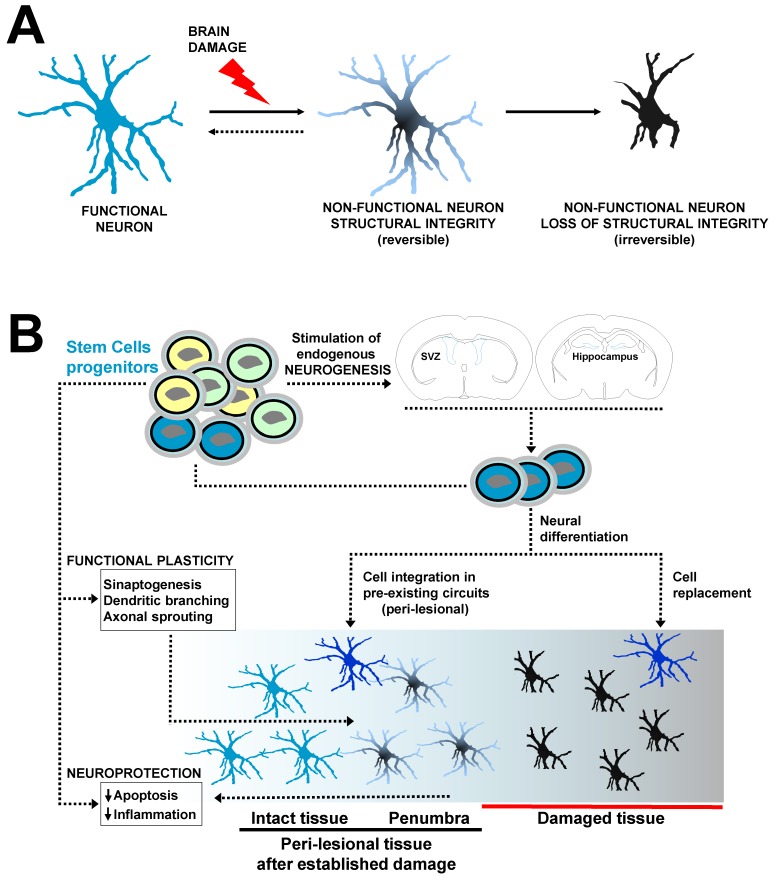
Stem cell mechanisms underlying recovery after cerebral damage. (**A**) After ischemic stroke, functional neurons (pre-ischemic; blue, left) go through different states including a reversible non-functional state called penumbra (black-blue, middle). If lack of blood flow persists, then irreversible neuronal death occurs (black, right). (**B**) Several mechanisms have been proposed to explain the therapeutic effects of different stem cells (SCs). (1) Neuroprotective mechanisms: At this level, the different SCs exert their effects by the reduction in apoptosis and diminishing of post-ischemic inflammatory response (immunomodulation). (2) Neuro-restitution and neuro-plasticity: The transplanted SCs might stimulate mobilization and production of endogenous neural SCs (stimulation of endogenous neurogenesis) or produce themselves different neural lineages, for example when exogenous nSCs are cerebrally implanted. All these neuro-therapeutic mechanisms induced by SCs might be drastically enhanced by engineered polymers which provide structural support for increasing survival and function of engrafted stem cells.

Over the last two decades, different type of cells (mostly multi- and pluripotent stem cells) have been used as therapeutic tools to enhance nervous tissue regeneration and brain plasticity phenomena (Figure 1). After the initial injury different SCs might neuroprotect the brain by the reduction in apoptosis and diminishing of post-ischemic inflammatory response that commonly exacerbates the brain damage propagating injury towards healthy tissue areas. The transplanted SCs might also stimulate the mobilization and production of endogenous neural SCs and progenitors in the subventricular zone (SVZ) and hippocampus (stimulation of endogenous neurogenesis) or produce themselves different neural lineages, for example when exogenous nSCs are cerebrally implanted. Exogenous SCs- or endogenous SCs-derived newborn neural cells (NNcells) might replace damaged cells creating new circuitry in the infarcted tissue or might be integrated in pre-existing neural networks in the perilesional tissue. Apart from NNcells integration, other forms of functional plasticity supported by the paracrine action of SCs (secretion of factors) occur at structural level, including the formation of new synapses (synaptogenesis), dendritic branching and growth and axonal sprouting favoring neuronal connectivity in the perilesional tissue, which have strong potential for plasticity after brain damage. In addition, different SCs may stimulate angiogenesis creating an optimal vascular network microenvironment for cell replacement, neural integration and plasticity. Many of these mechanisms can be favored by engineered polymers which provide structural and functional support for the engrafted stem cells.

Among the various types of stem cells, neural stem cells (nSCs) and neural precursors (nPCs) were initially established as the ideal cell populations for the repair of central nervous system. During embryonic development, neurogenesis through natural and pre-existing nSCs and nPCs principally occurs in two main brain regions: the subventricular zone (SVZ) and the ventral and lateral ganglionic eminences (VGE and LGE). The first region (SVZ) is the origin of the largest population of neocortical neurons while the ganglionic eminences give rise to the neurons of the basal ganglia and the neocortical interneurons [65]. In contrast, in the adult brain the main neurogenic niches are restricted to the ventricular/subventricular region as well as to the subgranular region (SGZ) of the dentate gyrus of the hippocampus [66]. Adult neurogenesis is regulated over several stages including maintenance and self-renewal of nSCs [67], proliferation and migration of nPCs, as wells as survival, maturation, and cellular integration in already established circuits or in ex novo built neural networks. After brain injury, endogenous nPCs migrate to the zones of damage [68,69] following the chemoattracting signals secreted by astrocytes, microglia, and vascular endothelial cells [66]. However, the number, recruitment and integration of neural cells are insufficient for the reestablishment of the neurological functions. Thus, although present, the endogenous neurogenesis is extremely inefficient which explains the lack of substantial post-stroke spontaneous recovery in humans and animals.

The implantation of nSCs has been considered a therapeutic option to favor the production and integration of terminal neural cells in either the core or the perilesional regions [70,71,72]. Additionally, donor nSCs stimulate endogenous neurogenesis [72,73] as well as angiogenesis [74]. Part of nSCs regenerative effects can be explained by their secretoma activity including the release of different proteins such as vascular endothelial growth factor (VEGF), brain derived neurotrophic factor (BDNF), nerve growth factor (NGF), and tumor necrosis factor alpha (TNF-α) among others [75,76]. To provide some representative examples of the therapeutic use of nSCs, in one study the implantation of human nSCs increased axonal transport, dendritic ramifications and axonal growth and propagation, all of these known to be mechanisms that contribute to the increase of neuronal plasticity, promoting the functional recovery of rats with focal ischemia [3]. 

In stroke mice, the intracerebral co-transplantation of human nSCs and the protease 3K3A-APC to stimulate the in vivo proliferation of transplanted nSCs reestablished the neural circuitry favoring post-stroke functional recovery [8]. However, when the nSCs were injected alone (without the 3K3A-APC protease), no significant recovery was observed, an effect that was in part related to a dramatic decrease in the injected nSCs content from two to three weeks after transplantation. This is a regular observation, since in many studies, and independently of the route of administration, the limited therapeutic effect of the transplanted SCs on post-stroke recovery may be ascribed with the poor engraftment and severe reduction of the transplanted cellular content in a hostile microenvironment as is the damaged brain [6,8,25,77]. As discussed later, a main role of biomaterials used for the integration of donor cells is to create an adequate structural microenvironment to favor the survival, retention, and cross-talk of the implanted cells with the host nervous tissue. Although nSCs-based regenerative therapies are very promising, the most significant disadvantages for their use in clinics are their scarce availability and difficulty of ex vivo expansion as well as the immunogenicity of allogenic transplants [78]. 

Embryonic stem cells (eSCs) constitute an alternative type of SCs that have been commonly used in a wide variety of studies. They are pluripotent cells capable of self-renewing and differentiating into any cellular phenotype of the organism including the neural lineage. The ability of eSCs to promote post-ischemic functional recovery has been demonstrated in several studies [79,80]. However the ethical limitations derived from its isolation from embryonic tissue [81] as well as the malignant transformation of this cellular phenotype and its tendency to frequently form teratomas in vivo [82] makes them less suitable for clinical applications.

Mesenchymal stem cells (mSCs), bone marrow mononuclear cells (BMmCs), and induced pluripotent stem cells (iPSCs) represent an alternative to nSCs and eSCs since they can be isolated, generated and expanded with no excessive difficulty. These cell populations have shown neuroprotective and neuro-regenerative effects in different animal models. They can be isolated or generated from the patients themselves therefore allowing autologous transplantation and avoiding graft rejection. Among the potential benefits of mSCs, iPSCs, and BMmCs it is worth mentioning the modulation of the brain tissue microenvironment through the secretion of several growth factors that regulate the immune response, limit astrogliosis and microgliosis, stimulate endogenous neurogenesis and angiogenesis or reduce apoptosis by decreasing the oxidative stress [83,84,85].

The iPSCs hold many of the functional properties found in eSCs, exhibiting similar morphology, the endogenous expression of pluripotency genes and their ability to self-renew or differentiate into progenitors, precursors, and mature cells of different germinal origin including neural cells [86]. Differentiated somatic cells can be reprogrammed to pluripotency (iPSCs) by treatment with defined factors. Several groups have been able to induce pluripotency easily and non-invasively from different somatic cells including fibroblasts, bone marrow, adipose tissue, and peripheral leukocytes [87,88,89]. Chen et al. found in a rat focal ischemia model that after the combined subdural transplantation of iPSCs and fibrin glue, there was a significant reduction in the infarct volume and a greater functional recovery in the animals examined with the rotarod test [90]. It has also been reported in infarcted rats that the intracerebral transplantation of iPSCs in both the affected and non-affected hemispheres induced their migration to the damaged area and subsequent differentiation into neural cells that enhanced post-stroke sensorimotor function recovery [91,92]. However, results are not conclusive since in other studies the iPSCs did not produce a substantial recovery of infarcted animals and the incidence of tumorigenesis was relatively elevated [93]. Thus, some limitations inherent to their genotype of iPSCs should be surpassed such as the possible mutations in cancer-related genes, aneuploidy and DNA aberrant methylation causing genomic instability, which have been associated with the formation of tumors and therefore incompatible with their clinical translation [93,94,95].

The BMmCs constitute a heterogeneous group of mature hematopoietic cells (B cells, T cells, and monocytes) and a small proportion of stem cells and hematopoietic progenitors, which together with stem cells and progenitors of mesenchymal origin (mSCs) represent a promising approach for the treatment of cerebrovascular disorders. BMmCs increase neurogenesis [62,96] and cerebral plasticity [97] as well as favor blood flow to the damaged tissue by increasing angiogenesis, thus creating a microenvironment suitable for the migration and integration of neural cells and the replacement of lost tissue promoting functional recovery in infarcted animals.

### 3.2. Mesenchymal Stem Cells 

mSCs are by far one of the most commonly employed cell populations. mSCs constitute a heterogeneous non-hematopoietic cell population which were first characterized by Friedenstein et al. in 1970 [98]. Although these cells can be isolated from different tissues including fat, umbilical cord and bone, it is in the bone marrow where they are present in a higher content [99], providing signaling for hematopoietic stem cell survival and function [100,101]. The special functional characteristics of mSCs and their strong potential for the treatment of cerebral disorders including stroke made them one of the better studied cell populations [35,36,37,102,103,104,105,106,107,108,109]. The wide variety of studies came through a significant advance in the knowledge of the neuroprotective and neurorepair mechanisms regulated by the paracrine activity of the mSCs. Pioneer studies reported mSCs are able to differentiate into neural phenotypes [110,111] but later studies have discredited the direct transdifferentiation of mSCs into neural lineages [112]. Current consensus is that most mSCs therapeutic effects are due to the secretome activity of these cells, which, independently of the administration route (cerebral, nasal, and intra-arterial/venous), is able to modulate the main neuroprotection mechanisms, i.e., post-stroke immunoresponse, inflammation and apoptosis [113].

mSCs may reduce inflammatory response and apoptosis by decreasing the levels of pro-inflammatory molecules such as interleukins IL-1β and IL-6A, or TNF-α [114,115,116]. Immunomodulation of the damaged brain is important to reduce the inflammatory response minimizing injury and promoting recovery [117]. Inhibition of inflammation has been related to the increased levels of neurogenesis [118]. mSCs participate in regulating the M1/M2 activation balance by biasing microglia differentiation towards the anti-inflammatory M2 state for resolution of inflammation and tissue repair in detriment of the pro-inflammatory M1 phenotype [119,120] and mSCs implantation has also been correlated with a reduced astrogliosis [121]. mSCs secretome also promotes angiogenesis, neurogenesis, and sinaptogenesis which in turn facilitate post-stroke brain repair and plasticity [122]. Angiogenesis in the perilesional tissue plays a crucial role in the survival and regeneration (restitution) of the damaged tissue. Intravenous administration of mSCs increases VEGF levels in rat ischemic tissue and provokes a strong development of microvasculature in the perilesional cortex of the infarcted hemisphere [102,123]. Angiogenesis as well as neurogenesis might be promoted by factors released by mSCs, including NGF, BDNF, VEGF, Glial-cell-derived-neurotrophic factor (GDNF), placental growth factor (PFG), or stromal-derived-factor (SDF-1) [2,6,102,124,125]. After ischemic damage, immature Doublecortine (DCX) positive neurons migrate from their neurogenic niches (SVZ) to areas of the perilesional tissue, deviating from their natural route to the olfactory bulb [126,127]. After thrombotic infarction in mice, the intracerebral transplantation of mSCs in the striatum was associated with increasing neurogenesis determined by an increment of cell proliferation and the content of DCX^+^ cells in areas surrounding the infarct core as well as with vascularization in the damaged area accompanied by a reduction of glial scar [6]. Finally, mSCs favor neuronal plasticity by increasing the number of synapses in both damaged and intact hemispheres in models of unilateral infarctions [128,129]. Axonal sprouting and myelin formation are essential for the reconstruction of the damaged neural circuits and could also be modulated by the implantation of mSCs in the brain [130].

Most of the above mentioned neurotherapeutic mechanisms are related to the factors released by the mSCs, which might have a certain parallelism with the secretome activity present in other types of stem cells with known neuroprotective and neuroregenerative potential. While many of these factors can be secreted by exocytosis which constitutes a main mechanism for releasing extracellular proteins or neurotransmitters in neurons, recent studies suggest that part of the paracrine action of mSCs may be accomplished by cell-derived vesicles also called exosomes that could transport mRNAs, miRNAs and proteins with tissue repairing potential [131,132]. In vitro studies have shown that exosomes stimulate the axonal growth of cortical neurons [133] as well as favor angiogenesis. Intravenous administration of mSCs in infarcted rats enhanced functional recovery through the microRNA 133b, which is present in exosomes released from mSCs [134]. In this study MiR-133b promoted axonal plasticity and neurite remodeling in the perilesional cortex [134].

### 3.3. Optimal Time Window and Best Administration Route

Although stem cell implantation in experimental models of stroke has been performed even several weeks after cerebral damage [61,135] significant post-treatment recovery is usually observed with cell implantation within 24 h to a few days after injury. This might be explained by the existence of a putative optimal intervention window for promoting recovery, neuro-restitution, and neuro-plasticity after brain injury consensually restricted to the first post-stroke days [5,6,8]. For example, physical rehabilitation is powerful when starts in the first 1–2 weeks after brain damage while no significant post-stroke improvement has been observed when started several weeks post-stroke [136]. However, many stroke patients continue improving for years after brain injury bringing to debate the limits of such temporal window. With respect to the SCs, there is no consensus regarding the best temporal point for their implantation [137].

The optimal route for cell implantation has still to be defined, although cerebral administration reports better benefits than that of intra-venous and intra-arterial infusion [137]. Surprisingly, the number of studies comparing the efficacy of different routes of administration using the same cellular phenotype and brain damage model is quite limited. In terms of therapeutic efficacy intra-arterial administration is comparable to intra-arterial infusion [138] and different types of SCs have been employed in models of cerebral infarction where, in many cases, functional improvement has been observed [102,104,114,138]. Although both routes of administration are relatively safe and less invasive than cerebral implantation, one main limitation of systemic intravascular transplantation is the reduced migration of the implanted cells towards the brain, which might be trapped in the pulmonary capillaries, spleen, kidney, or liver, thus diminishing its therapeutic effect [1]. In addition, systemic administration is not free of complications and risks, for example because of the formation of pulmonary and brain microembolisms that are mostly related to cell size and concentration as well as to infusion velocity [1,139,140]. In contrast, intranasal delivery of SCs is safe, feasible, and poorly invasive [141]. Cells administered via this route must pass across the intranasal mucous epithelium towards the brain tissue until they reach the area where therapeutic effect is needed [142]. However, the mucociliary tissue represents a barrier for particles and microorganisms where cells might get trapped, which obliges to increase the cellular doses to achieve a measurable therapeutic effect [142]. Up to date no clinical trials have been performed with this delivery route and it is unknown if the large migratory distance between mucociliary tissue and brain in humans represents a serious limitation [1].

Cerebral implants are more invasive than other transplantation routes. In general, the implantation of cells in the rodent brain has not been translated into important side effects. However, the human brain is extremely sensible to surgical manipulation and minor damages could create profound functional deficits beyond previous stroke-originated dysfunctions [143]. However, cerebral implants represent the most efficient approaches to promote post-stroke functional recovery [6,8,144,145]. They provide precision in terms of graft location as well as control of the number of the implanted cells. Furthermore, cerebral implants circumvent the blood–brain-barrier (BBB) which constitutes the stronger limitation for the transplanted cells when they are infused systemically or intranasally. The cerebral implants are also of relevance when hydrogels are used to assist the function of therapeutic cells. In a low percentage of patients SCs implantation has been related with important side effects such as focal hemorrhage, chronic subdural hematomes, seizures and psychomotor exacerbation [1,41,47,146]. However, cerebral implants might be justified by weighing the benefits of quality of life of the individual patient against the infrequent potentially life-threatening side effects. When considering the cerebral route there is also a lack of consensus regarding the best place for cell implantation to achieve a better recovery. Few cells survive beyond two or three weeks after cerebral transplantation [5,6,8,77,78], but the rate of survival is higher when SCs transplantation is performed in perilesional areas or far away from the cavity lesion [5,8]. After brain injury, the inflammatory response creates a hostile environment deleterious for both, the brain itself and the engrafted cells, making graft location critical for the survival of the implanted cells [5]. Short-lasting SCs survival inevitably leads to a non-durable therapeutic effect (cell differentiation and factors secretion). Similarly, if structural and functional changes of perilesional tissue are essential for plasticity and recovery, then SCs transplantation in these areas could be counterproductive. To overcome some of these obstacles, hydrogels-integrated SCs and factors have been implanted in the stroke cavity observing behavioral improvement in the animals treated despite a priori inhospitability of the infarcted area [144,147] or have been applied epi-cortically (to the brain cortical surface) in a theoretically less invasive approach [7].

### 3.4. Clinical Scenario: Where Are We Now?

The safety and tolerability of the implantation of SCs in patients with cerebral damage has been widely demonstrated for different types of SCs and for all administrative routes [38,39,40,41,42,43,44,45,46,47] although some adverse effects have been observed in a minor fraction of treated patients [41,47]. Results about the neuro-therapeutic efficacy of the SCs are not conclusive, possibly because of the small number of patients included in the studies and the variability of the location of the stroke. In one of the first clinical trials, Bang et al., proved both the safety and efficacy of the intravenous transplantation of autologous mSCs in stroke-injured patients. They reported a reduction of the neurological deficits in the large majority of the treated patients [38] but their conclusions raised concerns because of the reduced number of subjects and the lack of adequate controls [148]. Subsequent studies have demonstrated the safety and efficacy of the systemic administration of mSCs, here too, a reduction of post-stroke functional deficits was observed [44,149]. Recently, a comparison was made between the effect of intravenous injection of mSCs with respect to the combined co-transplantation of mSCs plus nSCs through the cerebellomedullary cistern [150]. After two-years follow-up, no serious adverse effects were observed (neurological infections, tumor formation) except for low fever (30% of patients) and temporary dizziness, and most patients showed clinical improvements [150]. However, the reduced number of patients (six subjects) limited the conclusions of the study. The safety and the therapeutic effects of systemic and intra-cerebral transplantation of BMmCs, nSCs, and mSCs have been corroborated by several other studies [41,46,47,151,152]. Collectively, there is a need of clinical trials involving a high number of patients, more homogenous and with better inclusion/exclusion criteria [153]. In addition, a greater number of pre-clinical studies are required to further investigate the neuro-therapeutic mechanisms of action of different SCs. The cellular and molecular substrates responsible for recovery are in general not well understood. In a few experimental studies, relevant mechanistic information for recovery has been obtained, which has been related to the production of nSC-derived newborn neurons and their functional integration with the host tissue [8], and structural changes (plasticity) related to axonal sprouting and dendritic branching of the host neurons after implantation with human neural progenitors and their secretome activity [3]. In contrast, the modest functional recovery observed in preclinical and clinical studies could originate from the cells implanted systemically and intra-nasally not migrating to the brain tissue with all the required efficacy [137]. In the case of cerebral transplantation poor recovery could be related to a progressive dispersion of the SCs outside the implanted area and to a higher mortality of the transplanted cells in the inflammatory post-ischemic brain tissue [5,6,77,124,154]. Poor engraftment of SCs into the brain is being tentatively resolved with different approaches, for example the combined implantation of SCs and factors that increase donor cell expansion [8] or the use of different biomaterials to assist cell survival and function [144], as we will discuss in the next section.

## 4. Hydrogels-Assisted Cell Therapies for Brain Stroke 

The use of biomaterials to assist cell therapy after brain injury is merely restricted to the experimental arena, which raises important concerns on whether we are really seeding a reasoned technology for future use in patients, or if we are facing an unreachable scenario. Preclinical data have supported the efficacy of the co-transplantation of hydrogels and cells to repair the damaged brain [135,144,155,156] and a growing number of clinical trials have demonstrated the feasibility and relative safety of cerebral implants with different SCs [39]. Thus, the available evidence envisages a horizon where different biomaterials, mostly in the form of hydrogels, could be useful in enhancing cellular engraftment to assist SCs therapeutic effects.

The concept of hydrogels for biomedical applications comes from the 1960s by using copolymers of glycolmethacrylates [157,158] to design structures with adjustable water content (>90% water), inertness with cells and tissues and permeable to metabolites through pores with adaptable sizes depending on polymerization conditions and crosslinking density. Hydrogels have been employed for a variety of biomedical uses such as for mimicking extracellular matrix tumors for cancer, for the delivery of chemotherapy drugs [159], or for heart repair [160]. Hydrogel polymers swell in contact with aqueous solutions and show a high affinity for absorbing water molecules remaining in an insoluble three-dimensional network state. Hydrogel properties can be adjusted to modify the gelation time or even delay their degradation both in vitro and in vivo. Various comprehensive reviews regarding the manufacturing methods of hydrogels and control of their properties have been included in the Reference Section [161,162,163]. Due to their high content of water hydrogels are ideal for implants in soft tissues such as the brain, in applications that span from cell encapsulation, to the control of the release of cell-secreted factors, but also for the controlled delivery of therapeutic drugs and different molecules, or just as imageable biomaterials [164]. In addition, hydrogels provide architectural support in the damaged and perilesional nervous tissue favoring cell-to-cell interaction and neural networks connections. Accumulated experience indicates that the mechanical properties of hydrogels modulate neural adhesion, proliferation differentiation and function. For example, it has been reported that neuronal adhesion on polyacrylamide gels increased with increasing elastic modulus, but this variation in stiffness did not modify neurite length [165]. In another study, the survival of neural cell cultures was better in more compliant hydrogels with compressive modulus below 3.8 kPa [166] or, more precisely, in the range of 0.1–1.0 kPa [167]. Stiffness can be modulated by changing the polymer concentration and the crosslinking density to achieve mechanical properties in the range of neural tissue [168,169,170]. In general, higher cross-linking densities and polymer concentrations lead to stiff hydrogels, while more compliant hydrogels are mostly obtained with fewer cross-links. The latter are supposed to be more adequate for brain applications [167]. Stiffness also decreases as a function of hydrogel degradation. It is generally assumed that matching the hydrogel mechanical properties with the brain stiffness is important to minimize contact stresses and to reduce the possible immune response. However, some controversy exists in the literature regarding the approximate stiffness of brain tissue, which probably is related with the analysis of mechanical properties in different anatomic regions, age and species used (human, primates, rodents) as well as with the testing methods (Table 1). For example, a mechanical stiffness of ~50 kPa has been determined in the mouse and rat brains by confined compression tests [170]. Alternatively, a brain tissue stiffness of 25 kPa has been found in the mouse brain using magnetic resonance elastography [169]. Other groups have reported lower compressive modulus for the brain tissue which range 10.0 kPa [166,171,172,173] to the lowest value of 0.3 kPa measured for the elastic modulus of cerebellum [174]. In addition to polymer concentration and crosslinking density, the stiffness of hydrogels can be tuned by changing parameters such as the molecular weight of monomers, the molar ratio of donor/acceptor groups, the number of reactive groups (i.e., acrylates in photo-crosslinked hydrogels) or by varying the ionic concentration [167,175].

Crosslinking is generally produced by physical non-covalent hydrophobic-ionic interactions and/or covalent bonds between chemical groups. The crosslinking-density and the relative amount of free and bound water is a main determinant of pore size and tridimensional structure. Natural and artificial hydrogels can be used alone or in combination. Naturally derived biomaterials are usually non-toxic; however, it is difficult to control their biodegradability and there are batch-to-batch variations in the production of the material that represent a source of variability in relation to their physical properties. Poor control of degradation in some natural polymers might be justified by the presence of degradation motifs such as hydrolysable ester groups and enzyme-mediated hydrolytic amide elements. An advantage of natural polymers is that they usually contain native binding sites to favor cell interaction and anchoring. Synthetic polymers in contrast have relatively well-controlled structures and their microstructure and properties including: pore size, degradation, functionalization, shape, sterilizability, and stiffness are adjustable. In comparison with natural materials, synthetic polymers might be created to mimic more efficiently the physical and mechanical properties of brain tissue. Synthetic polymers are usually non-inmunogenic, but may contain toxic products related to the fabrication and purification processes [189]. Generally, natural hydrogels might trigger host immune responses while synthetic ones no. To enhance biocompatibility and mimic the extracellular microenvironment, the hydrogel chemistry can be modified by adding different molecules and materials as for example gelatin, heparin or the tripeptide Arg-Gly-Asp (RGD), the principal integrin-binding motif present in matrix proteins such as fibronectin and some laminins and collagens to facilitate cell attachment and survival. Artificial hydrogels with degradable units such as poly(α-hydroxy acids) and poly(glycolic acid) can be synthetized, so that their constituent monomers dissociate in contact with water. Alternatively, other hydrogels, such as poly(ε-caprolactone) can be also enzymatically degraded by lipases.

One of the first studies using biomaterials for in vivo applications was performed in the 1980s [190]. Yannas and Burke performed skin implants in guinea pigs using collagen membranes and observed a limited inflammatory response by detecting mono- and polynucleated cells. This immune response against the implanted biomaterial could be minimized by crosslinking collagen with glucosaminoglucan. Later, Vacanti et al. cultured mouse and rat hepatocytes into artificial polymers composed of polyglactin, polyorthoesters, and polyanhydride for subsequent liver transplantation in rats, demonstrating that hepatocytes remained viable up to 14 days after in vivo implantation [191]. In the same period, a pioneer study showed prolonged in vivo survival and function of pancreatic islets encapsulated into cross-linked alginate microcapsules [192]. These studies provided some conceptual basis for the development of tissue engineering. Since then, a wide variety of applications in the field of regenerative medicine for different tissues and organs has been reported using hydrogel-based biomaterials to enclose different cells, drugs and different growth factors. 

### 4.1. Hydrogels for Brain Repair 

The first biomaterials for applications related to CNS disorders were assayed nearly three decades ago by Woerly et al., who intracerebrally implanted several materials with a base of collagen to enhance cellular attachment and provide pathways of migration for regenerating axons, transplanting a mix of polymers based on poly(glyceryl methacrylate) (pGMA) and poly(2-hydroxyethyl methacrylate)-collagen (pHEMA) in the cortex of adult rats. In their studies, astrocytes from the brain colonized the interior of the hydrogels. Although not immunophenotypically characterized, this cellular invasion responded to astrogliosis against the injection of the material in a formed hydrogel state and the nature of the materials used [193,194]. Lesny et al. replicated the Woerly assays using a similar composite of polymers (including pHEMA), confirming how hydrogels were colonized by reactive astrocytes up to eight weeks post-implant [195]. Thus, based on the ability of glial cells to enter inside these biomaterials, it was postulated that these polymers could be exploited in combination with different cell populations, constituting a viable approach to favor cell engraftment after implantation into the brain. Subsequently, poly-hydroxypropylmethacrylamide (HPMA) hydrogels were used to engraft eSCs and mSCs as imageable scaffolds for in vivo magnetic resonance in experimental models of spinal cord injury [196]. After this initial period, the use of biomaterials for neuroscience has continued to grow substantially over the years, providing examples of how the integration of cells and/or neurotrophic factors into biomaterials with different natures and composition enhanced the improvement of functional recovery in affected animals with respect to the therapeutic implantation of cells/factors without the biomaterial [13,14,144,197]. The different in vitro studies have provided support for the compatibility of distinct hydrogel-based biomaterials on the growth, differentiation, and function of different stem cells and progenitors as well the mechanistic aspects of differentiation and secretion of neuroprotective, neuroregenerative, and angiogenic factors [198,199,200,201,202,203]. However, the in vivo studies have provided a more definitive proof of the concept of the neuro-therapeutic potential of stem cells/progenitors [135,144,197,200,204] and different factors [7,205,206,207,208,209] cerebrally injected with different biomaterials. Any biomaterial of choice should provide a suitable microenvironment for the survival of the implanted cells, favoring cell engraftment in the host tissue [144,155,210,211]. Experimental data suggest that cellular behavior might be influenced by the nature of the biomaterial employed and the physical and chemical hydrogel properties [212]. For example, it has been described how extracellular matrix (ECM) stiffness affects stem cell maintenance, differentiation and function [213,214]. For cell engraftment in the brain parenchyma, an ideal hydrogel would be one with an adequate conformational structure and mesh size for cell lodging allowing the release of trophic factors, the entry of nutrients, and the output of waste products to create a barrier to protect therapeutic cells from a hostile microenvironment such as the damaged brain, because of the inflammatory and immune system response induced by injury. In addition, porosity of hydrogels can be tuned creating pores large enough to allow neurite extension and vascularization between the host brain and the implanted hydrogel [167]. As hydrogels are designed for soft tissues such as the brain, they are mechanically weak and difficult to handle in formed hydrogel state. However, hydrogels can be formed in situ under physiological conditions once a polymer (or mix of them) have been implanted as a liquid in the brain, making it easy to incorporate cells and factors before gelation, thus reducing the invasiveness of hydrogel implantation instead of using a formed hydrogel state [188,215]. This strategy allows, for example, the hydrogel to fill completely amorphous cavities as the result of injury, whereas formed hydrogels are not suitable for this type of applications. 

**Figure 2:**
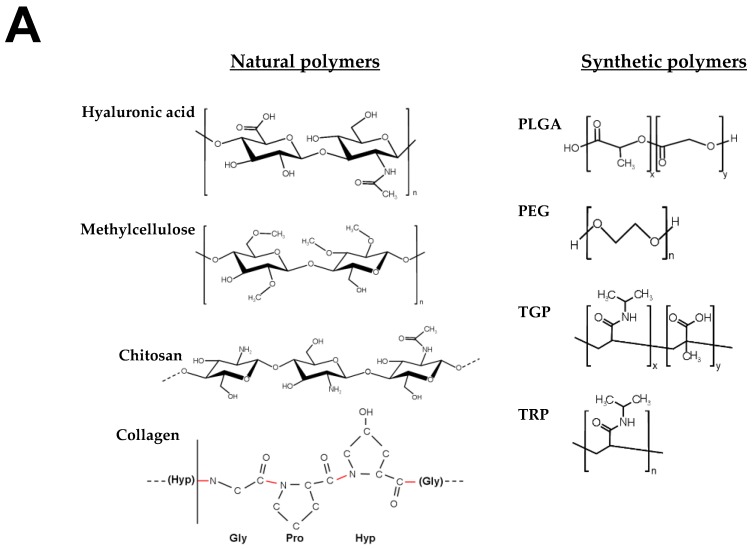

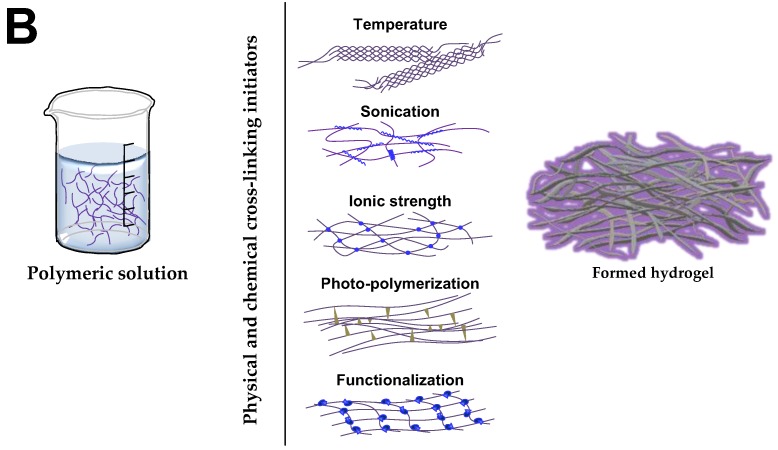
Hydrogel-based biomaterials for brain repair. (**A**) Chemical structure of the more frequent biomaterials and their constituent monomers used in neurorestorative therapies. (**B**) Hydrogel polymerization can be induced by physical and chemical cross-linking. Representative cross-linking initiators are temperature, electric/magnetic fields, ultraviolet exposition (photopolymerization), sonication, pH, ionic strength, solvent composition or functionalization among others. The cerebral implantation of hydrogel polymers (alone or in combination with cells) can be performed using the biomaterial in a formed state (polymerized before brain implantation) or injected as a liquid in the brain (pre-gel) to be formed in situ under physiological conditions. This latter approach reduces the invasiveness of biomaterial implantation allowing the hydrogel to fill amorphous cavities as the result of injury. PLGA: poly(lactic-*co*-glycolic acid); PEG: Poly(ethyleneglycol); TGP: poly(*N*-isopropylacrylamide-*co*-*n*-butyl methacrylate); TRP: poly(*N*-isopropyl- acrylamide).

In this regard, one of the basic design parameters of the hydrogel is the density of functional groups (i.e., acrylates, thiols, and polyesters) in the polymer backbone. The density of functional groups may influence the space between cross-links during polymerization, so that a higher density leads to hydrogels with a tighter mesh structure. Different strategies to induce hydrogel gelification have been developed for in vitro and in vivo applications [188,216,217]. Hydrogel polymerization can be prompted by changes in temperature, ionic concentration and pH. Alternatively, polymerization can also be induced by ultraviolet exposition (photopolymerization) or sonication [188] (Figure 2). An interesting property of certain hydrogel-based biomaterials depends on their ability to polymerize in situ at body temperature in certain concentration ranges. However, a major limitation of injectable hydrogels resides in the uncontrollable gelation kinetics. If gelation occurs too fast, obstruction of the needle can be produced during delivery. If the gelation is too slow, cells and factors enclosed into the biomaterial might disperse out of the brain site of implantation. In other scenarios the in situ polymerization does not result adequate, for example in peripheral nerve regeneration where axonal outgrowth probably needs biomaterial scaffolds with a pre-defined tubular shape to favor axonal guidance [218]. Once the neurotherapeutic effect has been concluded, hydrogels should degrade into non-cytotoxic and non-inflammatory by-products [219]. Often, degradation occurs by hydrolytic or enzymatic cleavage of bonds established between monomers during polymerization. Degradation might be useful for example to promote neurite extension from neural cells encapsulated into hydrogels at discrete sites of the hydrogel material allowing a certain control on the axonal sprouting and neural connectivity with the host tissue.

The combination of cells and hydrogels in pre-clinical models of neuro-restoration dates back to the last decade [220]. For the encapsulation of cells and factors, different natural and synthetic polymers alone or in a mix, have been manufactured including gelatin, polyethylene glycol (PEG), alginate, hyaluronic acid (HA), collagen, heparan sulfate proteoglycans, laminin, Matrigel, chitosan, polyacrylamide or PLGA, among others [221,222]. The possibility of integrating cells and factors into hydrogels for subsequent co-transplantation constitutes a strategy that may enhance cell survival, retention, and function as well as sustaining the delivery of different molecules when the material is injected within the stroke cavity, in peri-infarcted areas, epicortically, or in the brain striatum [7,156,181,183,206,209,210,223,224]. One of the most frequently used biomaterials for brain regeneration applications is hyaluronic acid, a high-molecular weight nonsulfated anionic glycosaminoglycan polymer composed of repeating units of β-1,4-d-glucuronic acid and β-1,3-*N*-acetyl-d-glucosamine (Figure 2). HA constitutes an abundant component of extracellular matrix in the brain, playing specific roles in cell adhesion proliferation and fate as well as providing signaling for wound repairing, angiogenesis or morphogenesis. To improve cell neural attachment and survival, HA hydrogels can be chemically modified with polylysine and RGD peptides [225]. In general, natural hydrogel polymers such as HA are more susceptible to suffer in vivo degradation via enzymes from the host tissue. For example the enzyme hyaluronidase, which is secreted by neurons and some glia, increases the rate of HA degradation in vivo. HA can be also degraded by ®-d-glucorinadases and ®-*N*-acetyl-hexosaminidases. Another material that has been used to a lesser extent for the treatment of neurological disorders is collagen, which represents a major structural component of extracellular matrix in various tissues. Functional collagen appears as a triple-helix and may lead to the formation of long fibers. Collagen can be also cross-linked to a variety of bioactive molecules by different physical and chemical procedures to favor interaction with cells and surrounding tissues. The sequences of amino acids Proline (Pro)-Hydroxyproline (Hyp)-Glycine (Gly) constitute the most common triplets (10.5%) in collagen (Figure 2). The natural biocompatibility of this biomaterial with many tissues represents a strong advantage for the design and fabrication of collagen hydrogels to support cerebral cell implantation. In contrast to the proteinaceous nature of collagen, chitosan is a natural polysaccharide produced by deacetylation of chitin formed by d-glucosamine and *N*-acetyl-d-glucosamine units (Figure 2). This biomaterial found in many arthropods is highly enriched in basic polysaccharides and has been used in a variety of different biomedical chitosan-based hydrogel applications. In addition, their mechanical properties can be tuned to mimic stiffness of many tissues including brain [187]. Alginate is a natural anionic polysaccharide extracted from algae composed of two monosaccharides of α-l-guluronic acid and β-d-mannuronic acid. The mechanical properties can be modified by the interaction of this biomaterial with divalent cations creating a dense 3D network. Intracerebral transplantation of alginate microcapsules has been used to neuroprotect the ischemic brain [226]. Several artificial polymers were also used for forming hydrogels in brain therapies. In this regard, PLGA is one of most commonly used biodegradable synthetic polymers in tissue engineering. PLGA is a linear copolymer that can be fabricated at different ratios from its main constituents: the cyclic dimers of lactic acid and glycolic acid. This biomaterial can be dissolved in a wide range of common solvents and be processed into any shape and size for encapsulation of factors, drugs and different cells. The chemical properties of PLGA allow hydrolytic degradation to produce the original monomers through de-esterification. PLGA degradation can be tuned by changing the ratio between monomers. This biomaterial has resulted very compatible in multiple applications including the functional restoration of damaged brain (Table 2). Additionally, PLGA microspheres at different rates based on PLGA carboxylation have been used to accelerate the release of different growth factors [227].

**Figure 3:**
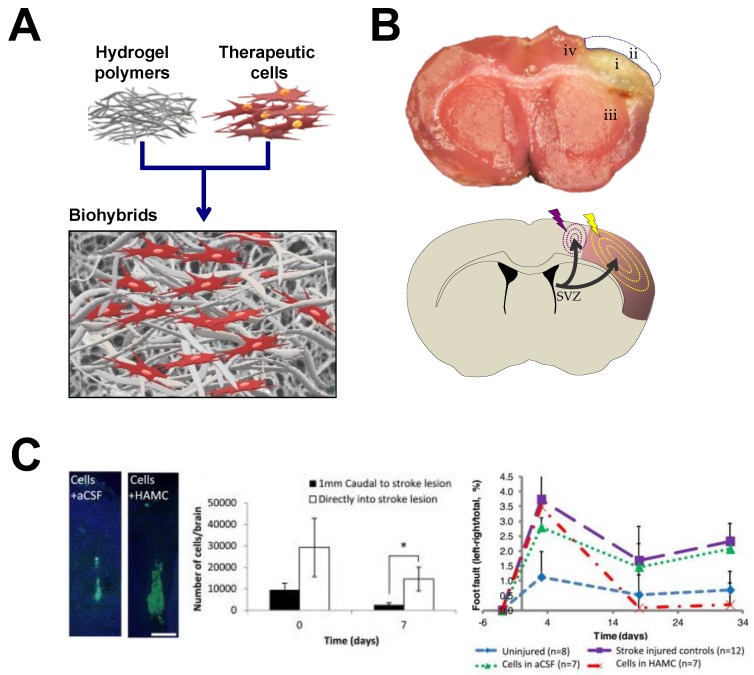
Neurorestorative potential of hydrogel-based biomaterials in ischemic stroke. (**A**) For encapsulation of cells and factors, different natural and synthetic polymers alone or in a mix have been manufactured and injected into different anatomic brain regions. (**B**) On the top, representative image of a coronal brain section stained with triphenyltetrazolium chloride (TTC) 24 h after distal middle cerebral artery occlusion, one of the stroke rodent models more regularly used [8]. In this model, the infarct area is mainly restricted to the brain cortex (“white” area in the right hemisphere) and the implantation of cells and hydrogels has been generally performed at different regions including: the stroke cavity (i); epicortically (ii); striatum (iii); or in the cortical area bordering the lesion cavity (iv). On the bottom, SCs might neuroprotect the brain by diminishing apoptosis and the post-ischemic inflammatory response. Furthermore, functional recovery induced by hydrogels and cells might be ascribed to regenerative processes in the damaged tissue (illustrated in the scheme by yellow dashed lines) or structural changes (tissue remodeling) in perilesional regions (violet dashed lines). Most of these changes are produced by previous stimulation of endogenous neurogenesis in the sub-ventricular zone (SVZ). (**C**) Example of the neuro-therapeutic potential of neural stem cells and progenitors (nSCs) integrated into hyaluronan-methylcellulose (HAMC) hydrogels. The engraftment and survival of nSCs into the mouse brain was favored when injected in HAMC hydrogels (left graph, scale bar 200 μm), with higher rates of survival when the cells enclosed in the biomaterial were injected into the stroke lesion (endothelin-1 model) than when they were implanted in healthy areas surrounding the damaged tissue (middle graph). The mice transplanted with nSCs delivered in HAMC hydrogels showed significant post-stroke functional recovery (right graph). Results in (**C**) reproduced with permission from [155], published by Cell Press, Elsevier Inc., 2015.

Several are the evidences supporting the therapeutic role of biomaterials, cells and factors for brain repair (Figure 3). In a representative study using a model of hypoxia-ischemia in mice, the intracerebral transplantation of nSCs cultured on scaffolds of polyglycolic acid (PGA) fibers (10–15 μm in diameter) inside the ischemic cavity resulted in axonal rewiring among the donor and host tissue with minimal adverse effects [204]. Although no functional evolution was examined, the study established a precedent in the co-transplantation of biomaterials and therapeutic cells in different models of cerebral damage. Later, HA hydrogels were constructed with laminin immobilized on the HA backbone and implanted in formed hydrogel state into the lesioned cortex of brain rats [178]. In this case the HA hydrogels were produced by cross-linking HA sodium salt with adipic dihydrazide (ADH) and carbodiimide, yielding elastic modulus below 1 kPa. These HA hydrogels functionalized with laminin sufficed to reduce astrogliosis and inflammation after injury as well as promote new vessels formation (angiogenesis) and neurite extension, a substrate for neuron-to-neuron connectivity [178]. The anti-inflammatory character of HA has been corroborated with other formulations based on this biomaterial [178,198]. For example HA hydrogels produced after chemical reaction with ADH derivates, were modified with anti-Nogo receptor antibody and poly-l-lysine and were embedded with PLGA microspheres functionalized with VEGF and angiopoietin-1 though water-in-oil-in-water emulsion techniques [198]. This HA-PLGA hydrogel composite was implanted in formed hydrogel state into the injury cavity of stroke mice attenuating the scar formation and the inflammatory response determined by the astrocytes content as wells as promoted angiogenesis and post-stroke behavioral improvement [198]. All these formulations led to hydrogels close to 1 kPa.

In other example, a high molecular HA modified with glycidyl methacrylate to create a photocrosslinkable HA polymer was used for spinal cord HA hydrogel implantation. In this case the compressive moduli reached a higher value of ~7.9 kPa. This value of the elastic modulus is similar to the value determined by the same group in the adult rat brain (5.7 kPa) and spinal cord (8.0 kPa) [177]. Once injected as a hydrogel this HA polymer reduced the inflammatory cell infiltration and gliosis on the surrounding tissue after spinal cord injury. In another study, a biodegradable porous gelatin-siloxane 3D scaffold formed by the integration of gelatin and 3-glycidoxypropyl trimethoxysilane was used to provide the gradual secretion of EGF/FGF neurothrophic factors [228]. When this material was implanted alone into the brain, the inflammatory response determined by the microglia content was minimal, thus supporting the biocompatibility of this biomaterial scaffold. The combined implantation of EGF/FGF with this biohybrid promoted tissue regeneration inferred by the accumulation of 5-Bromo-2′-deoxyuridine-proliferative cells, dendrite elongation, and endothelial vascular cell expansion, suggestive of active angiogenesis [228].

As a result of the promising outlook of the therapeutic approach, an increasing number of biomaterials in different formats have been assayed and extended in a wide variety of experimental brain damage models in the last decade (Table 2). Some of the most salient outcomes in neurorestorative therapies for stroke using advanced polymers in combination with different therapeutic cells are summarized below. For example, Jin et al. showed in rats that transplantation in the infarcted cavity of human eSCs-derived neuronal progenitors mixed with a suspension of liquid Matrigel for in situ gelation at body temperature improved sensorimotor, memory, and learning deficits after focal ischemia induced by occlusion of the middle cerebral artery [135]. Only the exclusive combination of cells and Matrigel was associated with a reduced cavity size, better survival of transplanted cells when integrated into the biomaterial and improvement of functional outcome. Although the experimental treatment was initiated three weeks after infarction, the behavioral improvement observed in the animals suggest that it is possible to rescue the brain damage at even longer times after injury, when the infarction and functional deficits has been definitively established and the inflammation is resolved [135]. In other study, injectable PLGA microparticles were fabricated through single oil-in-water emulsion technique and functionalized with allylamine and coated with fibronectin to provide structural support for nSCs. Once implanted into the stroke cavity new cerebral tissue was generated and cell morphology and behavior of transplanted cells was dependent on cell position relative to the infarct core, suggesting that the inhospitable core influenced on transplanted cells differentially with respect to the effect exerted by the perilesional tissue [156]. When these PLGA microparticles were modified with VEGF, endogenous endothelial cells chemo-attraction, migration and proliferation were observed to develop new vascular networks that might sustain the function of a de novo tissue within the stroke cavity [230]. A different combination of thiol-modified sodium HA cross-linked with heparin sulfate, collagen (gelatin) and polyethylene glycol diacrylate has been used to transplant eCS-derived neural progenitors into the lesion cavity of stroke mice [233]. This hydrogel matrix was able to polymerize at body temperature after brain injection. Although the mechanical properties of this biomaterial were not characterized in this study, it was shown that this mixture of polymers did not cause deformation of the brain after transplantation suggesting that the elastic properties were in the range of cerebral tissue. In addition, the hydrogel promoted the survival of transplanted cells and their differentiation into astrocytes and neurons in vivo. Another interesting finding was that the hydrogel alone diminished the post-stroke inflammatory reaction, a remarkable characteristic of HA-based biomaterials [233]. In yet another study, HA hydrogels with storage modulus in the range of 0.3–0.8 kPa favored the in vitro neural differentiation of iPSCs-derived nSCs [179]. In the in vivo context the same group used HA hydrogels functionalized through a Michael addition of acrylates present in the HA backbone with di-cysteins present in a matrix metalloproteinase degradable peptides [180]. These combined HA hydrogels presented a storage modulus of 0.3 kPa. When cerebrally injected in liquid format in combination with iPSCs-derived nSCs for in situ polymerization into the infarct cavity, the inflammatory response exerted by this HA-based biomaterial was minimal, in contrast to the greater inflammatory response observed after transplantation of HA polymers with storage moduli in excess of 1.0 kPa [180]. Thus, it is accepted that mechanical characteristics of hydrogels influence the degree of biocompatibility of the biomaterial and its integration into the host tissue [224], and stiffness properties close to the brain tissue are generally associated with lower inflammation and/or minor toxic effects. Despite this HA hydrogel caused minimal inflammation however it did not enhance survival of transplanted cells although significant neuronal differentiation was promoted [180]. Similarly, a fast (~10 min) in situ gelation polymer-based hydrogel composed of acrylated-HA, matrix metalloproteinase degradable and non-degradable motifs, adhesion peptides and heparin functionalized with thiol groups was used for cerebral implantation of iPSCs-derived nSCs [232]. This HA-hydrogel, with an elastic modulus in the range of 0.1–1.0 kPa, favored the survival of iPSCs-derived nSCs promoting glial and neuronal differentiation. By contrast, a copolymer of poly(*N*-isopropylacrylamide) and poly(ethyleneglycol) has been used to satisfactorily engraft mSCs into the mouse brain. This thermosensitive hydrogel with a sol/gel transition temperature of ~20 °C and storage modulus in the range of 0.5–1.0 kPa was implanted as a fully formed hydrogel over the brain cortex surface. Although no significant post-stroke recovery was observed after treatment in the interval of eight weeks of analysis, no significant inflammatory response induced by this specific copolymer was detected either [181]. In contrast, post-stroke functional recovery was efficiently enhanced in rats after epicortical transplantation of a another reversible temperature-responsive polymer (cell-sheet format) based on poly(*N*-isopropylacrylamide) in combination with mesenchymal progenitor stromal cells [231]. In another study with rats, nSCs were cerebrally transplanted together with porous 3D collagen sponges, which enhanced the survival of engrafted cells and synapse formation as well as improved neurological function after stroke [229]. An interesting observation with this study was that the collagen degraded completely thirty days after the biomaterial implantation, which might suppose a limitation with this biomaterial if persistent therapeutic stimulation is needed. Other combinations of biomaterials have been also tested. For example, a fast-gelling injectable in situ hydrogel mixture of HA modified with acetic hydrazide through carbodiimide chemistry and methylcellulose (MC), favored nSCs engraftment in the damaged brain, enhancing post-stroke motor recovery in an endothelin-1 mouse stroke model [155] (Figure 3). In addition, the transplanted nSCs differentiated into astrocytes and to a minor extent gave neurons and myelinating oligodendrocytes [155]. Although the mechanical properties of this hydrogel matrix were not assessed in this study, polymers with the same composition (HA and MC), but with slightly different proportions, showed elastic moduli inferior to 0.1 kPa [182]. In contrast, a blend of HA and MC injectable hydrogel, that was tested in the same stroke rodent model (endothelin-1), did not result in better survival of cerebrally transplanted iPS-derived neuroepithelial stem cells. It was hypothesized that the lack of a therapeutic effect might be attributable to the stresses produced by the flow of the cells along the needle, in addition to the extensional flow of cells in the transition syringe/needle during cerebral injection [200]. Very recently an interesting finding has been reported after the implantation of an advanced injectable porous HA hydrogel in the stroke cavity of infarcted mice after middle cerebral artery occlusion [183]. In a similar way to [180], this hydrogel was synthesized through carbodiimide chemistry to introduce acrylamide groups on the HA backbone. The biomaterial was functionalized with several peptides (including the RGD tri-peptide) and then cross-linked through Michael-type addition using di-cysteins present in matrix metalloproteinase degradable peptides giving a hydrogel scaffold with an elastic modulus of 1.5 kPa [183]. One difference with HA hydrogels of limited-porosity is that the implantation of this porous HA hydrogels was translated into a post-stroke reduced scar thickness, decreasing inflammatory microglia, and enhanced perilesional vascularization. In parallel, these porous HA gels stimulated the infiltration and migration of endogenous neural progenitors cells from the SVZ (neurogenic niche) towards the perilesional and stroke cavity regions, which could be related to an increasing infiltration of astrocytes [183]. Recently, a self-assembling peptide-based scaffold composed of repeating units of the IKVAV peptide sequence present in the major ECM protein laminin has been engineered [184]. This binding domain sequence (IKVAV) has been involved in neuronal adhesion, migration, proliferation differentiation and neurite guidance and outgrowth [234]. The IKVAV-injectable hydrogel with rheological elastic shear moduli below 1.0 kPa was implanted together with human eSCs-derived cortical progenitors into the lesion cavity of stroke rats (endothelin-1 model). This approach favored long-term survival of eSCs-derived cortical progenitors as well as promoted neuroprotection, cell replacement through the appearance of de novo tissue and supported cell adhesion and axonal growth, all of them mechanisms that contributed to enhance post-ischemia functional recovery [184]. The IKVAV sequence has been also previously used to functionalize HA-based polymers [185]. In this case, IKVAV peptides and metalloproteinase were immobilized to acryl groups of acrylated-HA through Michael type reaction giving HA hydrogels with elastic modulus in the range of 0.5–1.6 kPa. These copolymers were mixed with BDNF through electrostatic interactions to sustain BNDF release, and were also combined with human mSCs, enhancing nerve regeneration and recovery in a model of spinal cord injury in rats [185]. The biomaterial composition and stiffness of the implanted hydrogels not only affects the macrophages/microglia infiltration (a measure of inflammation) modifying the microglia M1/M2 balance [186], but also impacts on the in situ formation and retention of hydrogels in the stroke cavity, as it has been for example demonstrated in rats transplanted with an injectable ECM urinary bladder matrix-based hydrogel with storage modulus in the range of 0.07–0.4 kPa [186]. In this case, injection of higher ECM concentrations (>3.0 mg/mL) gelled within the stroke cavity, resulting in an extensive distribution and retention of the biomaterial within the cavity while reduced ECM concentrations were associated with permeation of ECM into the perilesional tissue [186,235]. Interestingly, when higher concentrations of this ECM hydrogel (>8.0 mg/mL) were injected into the stroke cavity, the biomaterial provided structural support to the surrounding healthy host brain tissue reducing the lesion cavity [235]. Collectively, these studies help us understand the importance of the design of a hydrogel with a defined structure and composition to provide a directed interaction with the cerebral microenvironment upon the pathological state resulting in minor damage and enhancing repair after injury. The applicability of stem cells and biomaterials has been tested in other neurological diseases of sudden onset, for example, traumatic brain injury (TBI). In rats, one week after TBI, the transplantation of a biodegradable injectable collagen gel favored donor mSCs survival and retention in the lesion core [236]. Although mSCs alone enhanced functional recovery, this effect was potentiated with the inclusion of this cell population in the biomaterial. In other example, the survival of nSCs was potentiated across eight weeks when this cell population was transplanted into the lesion cavity together with laminin/collagen- or fibronectin/collagen-based scaffolds with in situ gelation [237]. Similarly, this strategy induced progressive behavioral improvement after TBI in mice [237]. 

Across the different studies, the benefits of implanting SCs together with different biomaterials has been related to the modification (reduction) of post-stroke damaged areas; the formation of new vessels (angiogenesis) in the infarcted and peri-infarcted regions; or in the neuro-restitution and plasticity phenomena. In the case of angiogenesis, it is extensively accepted that vascular formation stimulates endogenous recovery mechanisms, creating an adequate microenvironment for endogenous proliferation migration and differentiation, thus supporting neural restitution, synaptogenesis, axonal growth, and neuro-plasticity [238]. These main therapeutic mechanisms are probably the result of the increasing survival and longer retention of implanted cells in the brain tissue, which is assisted by the different biomaterials used, favoring a persistent release of different neurotrophic factors responsible for the observed effects. Alternative approaches based on the direct implantation of different neurotherapeutic factors and hydrogels have been also developed and studied [239,240]. For example, two inducers of angiogenesis, VEGF and angiopoietin-1, have been implanted together with HA and PLGA in stroke models [198]. An enhanced stimulation of endogenous nSCs in parallel with the proliferation of neural progenitors have been found after the controlled release of cyclosporin A via a combination of HA and MC applied epicortically in rats submitted to stroke [7]. Post-stroke neurogenesis and axonal connectivity in the perilesional area promoted motor recovery after the controlled release of BDNF through HA hydrogels [206]. In another example, a hyaluronan/heparan sulfate hydrogel was used to control the delivery of antibodies against ephrin A, enhancing axonal sprouting and functional recovery [145].

### 4.2. Toxicity and Adverse Concerns

**Figure 4:**
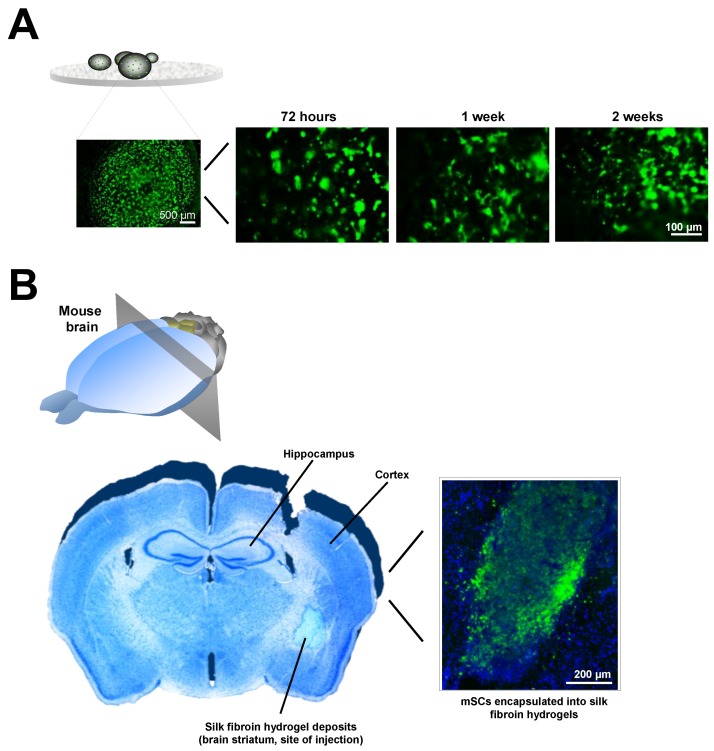
Stem cell engraftment assisted by silk fibroin-based hydrogels. (**A**) Positive staining for calcein (vital dye) showing how a specific stem cell population of mesenchymal origin (mSCs) survives in vitro integrated into silk fibroin hydrogel droplets during a two-week study period. (**B**) In vivo mSCs engraftment in the mouse brain is enhanced by the inclusion of this cell population into silk fibroin hydrogels [249]. Implantation of mSCs expressing the Enhanced Green Fluorescent Protein (EGFP) was done in the brain striatum of non-EGFP mice. In the figure, nuclei were stained with Hoechst (pseudocolor blue). It is known that the EGFP antigen might trigger an immunological response causing the loss of EGFP-cells in a non-EGFP brain microenvironment. However, the small pore size of silk hydrogels (~100–200 nm) seems to constitute a barrier to attenuate the immuno-attack of resident immune cells against the transplanted EGFP-mSCs. This barrier may also contribute to isolate and protect the engrafted mSCs from a hostile microenvironment as is the brain tissue after injury. Thus, this silk fibroin hydrogel favors the retention and survival of mSCs in the brain striatum for up to four weeks after transplantation (period examined in the image shown), a time frame that goes beyond the lifespan seen with different types of SCs engrafted without a biomaterial scaffold.

A deep reflection on the possible clinical applicability of the synthetic and/or natural biomaterials that are being used in the current brain damage models is necessary given that, with most biomaterials, the short- and long-term adverse effects derived from the implant into the brain are unknown. Many studies have focused on examining the therapeutic effects of a particular biomaterial alone or in combination with different cell populations without considering the analysis of additional cytotoxicity and inflammation induced by the material itself. In many cases, the compatibility of the biomaterials with brain-derived neural cell populations has been tested exclusively in vitro. However, in vitro studies offer a reductionist view of the tolerability of biomaterials with respect to in vivo approaches where it is possible to examine how the biomaterial can be integrated with the brain analyzing the host bioresponse towards the graft. For example, HA, one of the most frequently used biomaterials for experimental brain repair, does not produce significant astrogliosis, microgliosis, or increase neuronal death in the normal brain [145]. In fact, some tunable HA hydrogels with non-restricted permeability reduced the post-stroke inflammatory response [183]. However certain fragments of hyaluronan can trigger the innate immune response activating alloimmunity [241]. In this context, any biomaterial should be non-toxic both as a fully polymerized hydrogel and as any by-product that may be generated during its degradation. In this regard, the accumulation of HA has been related to aging processes and demyelinating diseases such as multiple sclerosis. Some forms of hyaluronan produced by astrocytes accumulate in demyelinated lesions avoiding the remyelination of neuronal circuits after brain damage [242,243]. Although a clear positive effect of HA promoting post-stroke recovery (including the reduction of inflammation) has been reported, reasonable doubts emerge with the usefulness of this biomaterial for application in other neurological disorders when considering the clinical perspective. As discussed previously, other biomaterials with proven neuro-therapeutic potential are collagen, chitosan, PLGA, or Matrigel. However, the use of collagen as a biomedical implant raises safety concerns related to the possible contamination of this biomaterial with viruses and prions, the latter related with neurodegenerative diseases such as bovine spongiform encephalopathy. Collagen is difficult to obtain due to its limited sources and can be degraded easily, which is a handicap if a prolonged neurotherapeutic effect is needed. PLGA degrades into by-products that might exacerbate inflammation, therefore producing additional brain damage after brain injury [244,245]. In the case of chitosan, this biomaterial shows fast degradation and some preparations cause allergic reactions due to residual components [246]. Matrigel, a solubilized mix of ECM proteins extracted from the Englebreth-Holm-Swarm mouse sarcoma, is not adequate for clinical application despite the proven experimental therapeutic potential of nSCs embedded in this material to enhance post-stroke functional improvement [135]. Fibrin hydrogels might support cells adhesion and growth; however, the poor stiffness, fast degradation, and shrinkage of this biomaterial represent a limitation for the clinics. In this context, silk fibroin constitutes an interesting biomaterial for stem cells/factors therapy in brain injuries and neurodegenerative diseases. This flexible and adaptable biomaterial has been used for years in a variety of biomedical applications [247,248]. We have recently developed an in situ fast-gelling silk fibroin hydrogel through the sonication-induced of regenerated silk fibroin solutions [188]. This hydrogel, with elastic modulus in the range of 6–30 kPa [188], favors stem cell survival in vitro and in vivo (Figure 4) [249] and can be injected in the brain tissue without causing significant inflammatory or neural death responses in an interval of four weeks of analysis [188]. In our specific study, after brain-silk-fibroin transplantation, we did not find impairments of sensorimotor and cognitive capacities or sleep-wake disturbances [188]. However, we do not have any evidence regarding whether this silk fibroin biomaterial might be therapeutically more efficient than other alternative biomaterials. From our point of view, in terms of clinical applicability, the in vivo biocompatibility of different materials with brain tissue should not be checked exclusively by the examination of tissular inflammatory or cytotoxicity responses without addressing important aspects of brain functionality. For example, it would be widely recommended to perform animal behavioral testing to examine possible sensorimotor, memory, and learning abnormalities as a consequence of the biomaterial implantation or even to further the study of structural and functional connectivity by using anatomic tracers, electrophysiology, and brain imaging-based approaches [188,235]. In contrast, there are also a very limited number of studies comparing the in vivo toxicity of different biomaterials as well as the therapeutic efficacy under the same brain damage models. In this context one would expect a comparison between different biomaterial candidates which are under study for brain repair following brain damage. However, no sufficient effort has been made in this sense creating a lack of consensus with respect to which biomaterials look most promising. In one in vitro study it was found that nSCs migration, proliferation and differentiation were better supported by laminin matrices and Matrigel than fibronectin or poly-l-ornithine [250]. In other example, a comparison between different HA and PEG hydrogel formulations was undertaken to examine the survival, growth and differentiation of neural progenitor cells [251]. We do not have enough information regarding both the optimal site of cerebral implantation and temporal point of intervention to achieve the best cell-biomaterial engraftment/therapeutic effect compromise after brain injury. Post-stroke recovery has been largely demonstrated in a majority of studies implanting cells and biomaterials but functional outcome was evaluated with different readouts and distinct brain damage models being unknown which therapeutic conditions might be more favorable [56]. In most cases, the molecular and cellular mechanisms responsible for recovery after brain cell transplantation have not been completely elucidated. Regarding the action mechanisms of SCs a nice contribution has been recently made by Wang et al. They demonstrated how sensorimotor recovery after stroke was due to the structural and functional integration of transplanted nSCs with the host brain circuitry examined by voltage-sensitive dye imaging and anatomic tracers [8].

## 5. Concluding Remarks 

Cures for stroke and other CNS injuries through engineered biomaterials will have a major positive impact in heavy and long-term burdens on patients and healthcare systems. The development of advanced biomaterials for cerebral damage responds to a major clinical need to provide patients with an efficient technological solution. Across the last two decades of experimental research, it has been possible to partially restore functionality loss after brain injury by using biomaterials of different composition and format to favor the engraftment of cells with neuro-therapeutic abilities. Despite the valuable contribution of behavioral studies in examining the improvement of functional outcomes in animals, in many cases, the molecular and cellular substrates underlying recovery are unknown, making it necessary to further investigate these mechanisms to rationally identify those targets whose modulation through cells and biomaterials might lead to greater therapeutic benefit. In addition, it would also be ideal to advance the screening of those more promising biomaterials such as those that are poorly inmmunogenic and less cytotoxic with controlled stiffness properties to facilitate their integration with the brain tissue through minimally invasive procedures. Tolerability of hydrogels should be examined using a multidimensional approach that could initially include preliminary in vitro studies, and the subsequent implantation in animals to analyze the in vivo bio-response at both the structural and functional level. It is also essential to examine concerns regarding the best temporal point of intervention and site of implantation to achieve an efficient post-brain-damage therapeutic response.

Although cerebral implantation of biomaterials-based hydrogels has not been applied in human subjects after brain injury, the antecedents presented in the preclinical (stem cells plus biomaterials) and clinical (stem cells) scenarios outline a magnificent opportunity for the treatment of CNS disorders using distinct biomaterials. In recent years, the fast evolution of this field predisposes the continuation of the search, characterization, and improvement of advanced hydrogels to clinical translation.

## Figures and Tables

**Table 1:** Mechanical properties of brain tissue and hydrogel polymers used for cell encapsulation in neurorestaurative therapies studies.

**Organ/tissue**	**Measured property**	**Measured value**	**References**
Brain (rat and mouse)	Elastic modulus (Compressive)	E = 50 kPa	[170]
Brain (rat)	Shear storage modulus	G′ = 0.33 kPa	[171]
Brain (rat)	Shear storage modulus	G′ = 0.6 kPa	[172]
Brain (swine)	Elastic modulus	E = 3.2 kPa	[176]
Brain (mouse)	Shear modulus	E = 25 kPa	[169]
Brain (rat)	Bulk elastic modulus	E = 5.5 kPa	[173]
Brain (rat)	Bulk elastic modulus	E = 5.7 kPa	[177]
Cerebellum (rat)	Bulk elastic modulus	E = 0.3–0.45 kPa	[174]
**Biomaterial hydrogel**	**Measured property**	**Measured value**	**References**
PEG	Elastic modulus	E = 1–10 kPa	[166]
HA-Laminin	Shear Storage/Loss modulus	G′ (Storage) = 0.6 kPa G′′ (Loss)= 0.2 kPa	[178]
HA	Bulk elastic modulus	E = 7–8 kPa	[177]
HA	Shear Storage/Loss modulus	G′ = 0.33–0.84 kPa G′′ = 0.001–0.01 kPa	[179]
HA with MPP degradable	Shear Storage/Loss modulus	G′ = 0.33 kPa G′′ = 0.0057 kPa	[180]
HA with MPP nondegradable	Shear Storage/Loss modulus	G′ = 0.29 kPa G′′ = 0.0024 kPa	[180]
TGP (Mebiol gel)	Shear Storage/Loss modulus	G′ ~ 0.8 kPa (~37 °C) G′′ ~ 0.2 kPa (~37 °C)	[181]
HAMC	Shear Storage/Loss modulus	G′ < 0.1 kPa G′′ < 0.1 kPa	[182]
Microporous HA	Elastic modulus	E = 1.5 kPa	[183]
Laminin peptide sequence IKVAV	Shear Storage/Loss modulus	G′ = 0.8–1.0 kPa G′′ = 0.1–0.4 kPa	[184]
HA-IKVAV-MPP	Shear Storage/Loss modulus	G′ = 0.47–1.6 kPa G′′ < 0.1 kPa	[185]
ECM-UBM	Shear Storage/Loss modulus	G′ = 0.07–0.46 kPa G′′ < 0.01–0.06 kPa	[186]
Chitosan	Shear Storage/Loss modulus	G′ = 0.8–1.5 kPa G′′ = 0.001–0.01 kPa	[187]
Alginate	Shear Storage/Loss modulus	G′ = 0.8–1.5 kPa G′′ = 0.4–0.5 kPa	[187]
Silk fibroin	Elastic modulus	E = 6–30 kPa	[188]

PEG: Poly(ethyleneglycol); PLGA: poly(lactic-*co*-glycolic acid); HA: hyaluronic acid; HAMC: blend of hyaluronan and methylcellulose; TGP: thermoreversible polymer block poly(*N*-isopropylacrylamide-*co*-*n*-butyl methacrylate); MPP: metalloproteinase; IKVAV: Ile-Lys-Val-Ala-Val peptide; ECM-UBM: extracellular matrix composed of urinary bladder matrix-based hydrogel.

**Table 2:** In vivo studies using different cell populations in combination with distinct biomaterials to restore the functionality loss after brain stroke.

Stroke model, specie	Biomaterial, cell population, site of implantation	Therapeutic effects	References
CCAO, mouse	PGA, nSCs, infarct cavity	Increasing axonal rewiring, reduction of inflammation and glial scar formation	[204]
MCAO, mouse	TGP, mSCs, brain surface	Increasing engraftment of transplanted cells, increasing neuronal differentiation, no functional improvement	[181]
MCAO, mouse	HA alone, infarct cavity	Reduction of inflammation and glial scar, enhanced perilesional vascularization, stimulation of endogenous neurogenesis	[183]
MCAO, rat	PLGA, nSCs, infarct cavity	Cavity size reduction, increasing engraftment of transplanted cells, de novo tissue formation	[156]
MCAO, rat	Matrigel, eSCs-nPCs, infarct cavity	Reduction in lesion size, increasing survival of transplanted cells, neuronal and astroglial differentiation, neuronal migration, improvement of behavioral outcome	[135]
MCAO, rat	Col I , nSCs, infarct cavity	Increasing survival of transplanted cells, neuronal differentiation, increasing synaptogenesis, improvement of behavioral outcome	[229]
MCAO, rat	PLGA-VEGF, nSCs, infarct cavity	Hipervascularization linked with astrocytic differentiation, limited neuronal commitment	[230]
MCAO, rat	TRP, mSCs, brain surface	Improvement of motor function	[231]
PTS, mouse	HA, iPSCs –nPCs, infarct cavity	Enhancing survival of transplanted cells, reduction of post-stroke inflammation, increasing neuronal and astrocytic differentiation	[180,232]
PTS, mouse	HA-heparin-Col, eSCs-nPCs, infarct cavity	Increasing survival of transplanted cells, reduction of inflammation and glial scar	[233]
ET-1, mouse	HAMC, nSCs, infarct cavity	Increasing survival of transplanted cells, astrocytic differentiation, limited neuronal and oligodendrocyte commitment, improvement of behavioral outcome	[155]
ET-1, rat	Self-assembling IKVAV peptide (laminin epitope), eSCs-nPCs, infarct cavity	Increasing survival of transplanted cells, tissue regeneration, cell adhesion and axonal growth, improvement of behavioral outcome	[184]

CCAO: common carotid artery occlusion stroke model; MCAO: middle cerebral artery occlusion stroke model; PTS: Photothrombotic stroke model; ET-1: endothelin-1 stroke model; PGA: poly(glycolic acid); PLGA: poly(lactic-*co*-glycolic acid); Col I: collagen type I; HA: hyaluronic acid; HAMC: blend of hyaluronan and methylcellulose; TGP: thermoreversible polymer block poly(*N*-isopropylacrylamide-*co*-*n*-butyl methacrylate); TRP: thermoreversible poly(*N*-isopropyl- acrylamide); IKVAV: Ile-Lys-Val-Ala-Val peptide; nSCs: neural stem cells; nPCs: neural progenitor cells; mSCs: mesenchymal stem cells; iPSCs: induced pluripotent stem cells; eSCs: embryonic stem cells; VEGF: Vascular endothelial growth factor.
